# COLLABORATIVE RESEARCH WITH A MIXED-METHOD DATASET USING DECEDENT DATA

**DOI:** 10.1093/geroni/igad104.0987

**Published:** 2023-12-21

**Authors:** Kelly Melekis, Carol Weisse

**Affiliations:** University of Vermont, Burlington, Vermont, United States; Union College, Schenectady, New York, United States

## Abstract

This presentation will present opportunities and challenges in building and sharing a mixed method dataset of terminally ill hospice patients from residential homes for the dying (social model hospice). As nonprofit, community-based settings, residential homes for the dying document care decisions using paper files and handwritten notes, and files range in size based on length of stay (with some files being hundreds of pages in length). Through a multi-site community-university partnership, we have scanned over 500 patient records, extracted quantitative data, and de-identified data for qualitative analysis. We will share the challenges of data management and organization, extraction, and de-identification. We will also discuss ethical issues related to working with decedent data, community-engaged research, and multi-site collaboration. Based on our experiences with qualitative data analysis of resident files, we will share strategies for extracting qualitative data from decedent records, using qualitative data analysis software, and sharing data for collaborative research.

